# Vulvar Carcinoma in Pregnancy: A Case Report on Diagnosis, Management, and Surgical Outcomes

**DOI:** 10.7759/cureus.76611

**Published:** 2024-12-30

**Authors:** Anitha Reddy, Swathi Donoori, Kalidindi Venkata Vijaya Narsimha Raju, Tejashwini Pogu

**Affiliations:** 1 Obstetrics and Gynaecology, Fernandez Hospital, Hyderabad, IND; 2 Surgical Oncology, Basavatarakam Indo American Cancer Hospital and Research Institute, Hyderabad, IND; 3 Obstetrics and Gynaecology, Fernandez hospital, Hyderabad, IND

**Keywords:** biopsy, histopathological examination (hpe), lichen sclerosus, pregnancy, vulvar carcinoma, vulvectomy

## Abstract

Vulvar cancer is an uncommon malignancy in reproductive-aged women, and its occurrence during pregnancy is rare. This report presents a case of vulvar squamous cell carcinoma (VSCC) diagnosed perioperatively in a 35-year-old pregnant woman. The patient was incidentally found to have lichen sclerosis and a suspicious vulvar lesion during an emergency cesarean section. Biopsy confirmed VSCC, and subsequent management involved a simple vulvectomy, sentinel lymph node biopsy, and local advancement flap reconstruction. Histopathological examination revealed clear margins and no lymph node involvement. This case highlights the importance of routine external genital inspection during pregnancy to enable early detection and timely management of vulvar lesions.

## Introduction

Vulvar cancer is an uncommon malignancy accounting for approximately 4% of all gynecological cancers. With a global incidence of 45,240 new cases annually, vulvar squamous cell carcinoma (VSCC) is the most common histological subtype [1]. While vulvar cancer predominantly affects postmenopausal women, the human papillomavirus (HPV) associated form occasionally presents in younger women of reproductive age. Chronic inflammatory conditions such as lichen sclerosus also predispose women to vulvar cancer typically in women in their 70s [2]. During pregnancy physiological changes can obscure symptoms delaying diagnosis and treatment. Early identification and multidisciplinary management are critical to optimizing maternal and fetal outcomes.

## Case presentation

**Figure 1:**
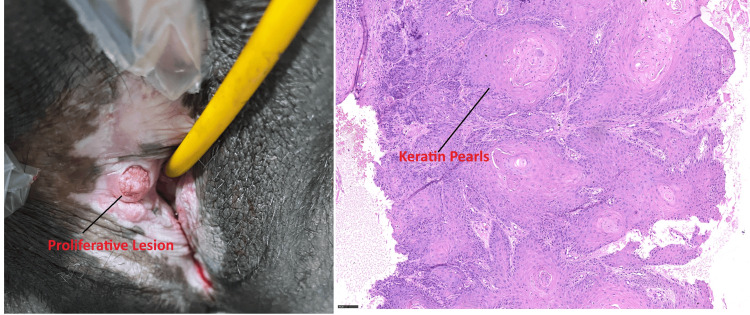
Proliferative lesion on the right side of the labia minora and histopathological examination (HPE) of the lesion The left side image shows lichen sclerosis and exophytic proliferative lesion. The right side image shows the histological section of squamous cell carcinoma, showing nests of malignant squamous cells with prominent keratin pearls and stromal invasion.

**Figure 2:**
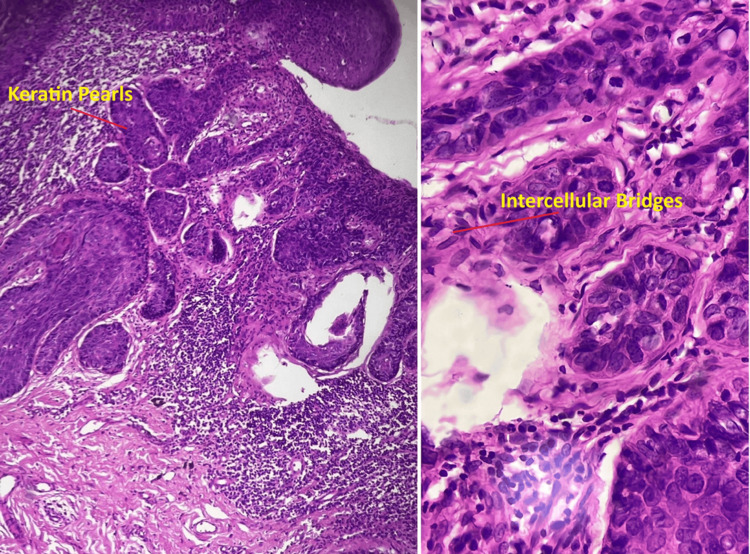
Histopathological examination (HPE) of the vulvectomy specimen Low magnification (left-side image): shows invasive nests of malignant squamous cells with prominent keratin pearls characteristic of squamous cell carcinoma. High magnification (right-side image): shows intercellular bridges,a hallmark feature of squamous  differentiation further confirming the diagnosis.

A 35-year-old woman, gravida 3 para 1 living 1 abortion 1 (G3P1L1A1) with a previous cesarean section, was registered for care at eight weeks. All baseline investigations were within normal limits. Her antenatal period was uneventful. Maternal and fetal monitoring was done as per the hospital protocol. At 35 weeks of gestation, a routine scan revealed severe oligohydramnios with an amniotic fluid index (AFI) of 4.5 cm. The fetal weight was appropriate for gestational age, and Doppler studies were normal. Considering the severity of the condition and potential risks to fetal well-being, the patient was admitted and underwent an emergency lower segment cesarean section (LSCS). During the perioperative period, lichen sclerosus was observed involving the entire vulva. In addition, a suspicious proliferative lesion measuring 2 x 2 cm was noted on the right side of the labia minora, located 1 cm lateral to the clitoris (Figure 1). She underwent an emergency cesarean section and birthed a baby girl weighing 2300 grams with a good Apgar score. Upon questioning, she reported experiencing an itching and burning sensation over the vulva for the past few weeks. Post cesarean section, detailed counseling was done by the team, and a biopsy of the lesion was taken after obtaining consent from the couple and sent for histopathological examination to rule out malignancy. Histopathological examination of the biopsy specimen revealed the presence of well-differentiated squamous cell carcinoma (Figure 2). The patient was subsequently referred to a cancer hospital for further evaluation and management.

Metastatic evaluation was conducted using clinical examination and ultrasound, revealing the presence of enlarged lymph nodes in the right inguinal region, without other evidence of metastatic disease elsewhere in the body. Fine-needle aspiration cytology (FNAC) of the right inguinal node was performed, which showed no evidence of malignancy. She was clinically staged as cT1N0 M0. After a thorough discussion with the family regarding the pros and cons of the surgical options, a decision was made to proceed with a simple vulvectomy. On the 10th day of the post-cesarean section, the surgical procedure began with a simple vulvectomy, which involved the removal of the affected vulvar tissue. This was followed by a local advancement flap reconstruction to repair and close the surgical site, ensuring better aesthetic and functional outcomes. During the surgery, a sentinel lymph node biopsy was also performed. The sentinel lymph node biopsy involved the removal of the first lymph node or group of nodes to which cancer cells are most likely to spread from the primary tumor. This biopsy was sent for a frozen section analysis during the operation, which fortunately returned negative for malignancy.

Post-surgery, the histopathological examination (HPE) of the vulvectomy specimen revealed squamous cell carcinoma with all margins clear of the tumor, confirming that the cancer had been completely excised. The final staging of the tumor was determined as PT1b N0(sn)M0. This staging is significant as it denotes a good prognosis for the patient, with a high likelihood of successful treatment and a low risk of recurrence. The comprehensive surgical approach, including the clear margins and negative sentinel lymph node biopsy, underscores the effective management of the patient's condition, aiming for optimal recovery and minimal complications. She was discharged on the third postoperative day in stable condition.

The patient was educated about the importance of regular follow-up visits. She was scheduled for follow-ups every three months for the first three years. During each visit, she underwent a physical examination, pelvic examination, and Pap test, all of which were normal. One year post-surgery, the patient remains in good health with no evidence of disease recurrence.

## Discussion

Vulvar cancer is an uncommon malignancy, accounting for a small percentage of gynecological cancers, and its occurrence during pregnancy is rare. This presents unique challenges in diagnosis and management due to the physiological changes associated with pregnancy and the need to balance maternal health with fetal well-being.

Epidemiology

Vulvar cancer typically affects postmenopausal women, with the average age of onset being in the mid-60s. The occurrence during the reproductive years is rare, and during pregnancy, it is an exceptional finding. This rarity contributes to limited literature and case studies, resulting in challenges in formulating standardized treatment protocols [1].

Pathophysiology and Risk Factors

Vulvar cancer primarily involves squamous cell carcinoma, although other types, such as melanoma, can also occur. Risk factors include chronic conditions like lichen sclerosus, human papillomavirus (HPV) infection, immunosuppression, cigarette smoking, and a history of precancerous lesions. During pregnancy, hormonal and immunological shifts can theoretically impact the progression of preexisting lesions, although direct causality remains underexplored due to the rarity of the condition [2,3].

Clinical Presentation and Diagnosis

The diagnosis of vulvar cancer during pregnancy can be delayed due to overlapping symptoms with common pregnancy-related conditions such as vulvar itching, swelling, or discomfort. Suspicious signs include persistent lesions, ulcerations, or nodular growths on the vulva. Definitive diagnosis is established through a biopsy and histopathological examination. In our case, a biopsy was performed after identifying a suspicious lesion on the vulva [4].

Management

Treating vulvar cancer during pregnancy requires a multidisciplinary approach involving gynecologic oncologists, obstetricians, and neonatologists. The timing of the diagnosis within the pregnancy significantly influences management decisions:

First and second trimesters: If detected early, surgical excision is often considered, taking into account the safety of the fetus and maternal outcomes.

Third trimester: Treatment may be postponed until after delivery unless urgent intervention is required. 

Surgical management, such as wide local excision or vulvectomy, is typically the primary approach. Adjuvant treatments like radiation and chemotherapy are generally avoided during pregnancy due to potential teratogenic effects, although carefully weighed options may be considered if the cancer is advanced and poses a significant risk to the mother [5,6]

Prognosis and outcomes

The prognosis for vulvar cancer diagnosed in pregnancy largely depends on the stage at diagnosis. Early-stage cancers that are treated promptly can have favorable outcomes for both mother and child. Delays in diagnosis, however, may lead to more complicated treatment courses and impact maternal prognosis. Close monitoring and individualized treatment planning are essential to optimize outcomes [7,8].

In the current case, the woman did not initially report any history of burning or itching, as she believed these symptoms were not significant.

Educating women about the importance of providing a complete history of any vulval or genital lesions is essential for early detection and effective management during pregnancy. Many women may not realize the significance of reporting past or present lesions, such as warts, sores, or unusual growths, as part of their gynecological history. Therefore, healthcare providers should emphasize the following points

Open Communication

Encourage women to be open about any past or current genital issues, including itching, pain, swelling, or visible lesions, as this information can help healthcare providers assess risks and provide the best care.

Potential Impact on Pregnancy

Explain how certain infections or conditions, like herpes or genital warts, could affect pregnancy outcomes or the mode of delivery (e.g., the need for a Caesarean section in some cases).

Early Intervention

Highlight that reporting lesions early allows timely treatment, potentially reducing the risk of complications during pregnancy and birth.

Follow-Up Care

Reinforce the need for regular follow-up, especially if they notice any new changes in the genital area, even after the initial consultation.

Routine local inspection of the external genitalia during the first trimester of pregnancy can help in the early detection and management of vulvar lesions. 

## Conclusions

Vulvar cancer during pregnancy remains a rare and complex clinical scenario requiring careful consideration and multidisciplinary care. Further research and documentation of cases are crucial to improve understanding and guide management strategies, ensuring the best possible outcomes for both the mother and the fetus. Educating women in these areas empowers them to actively participate in their healthcare additionally routine examination of the external genitalia in the first trimester of pregnancy helps better management and prevention of complications related to vulvar lesions during pregnancy.
